# MeCP2 Mutation Results in Compartment-Specific Reductions in Dendritic Branching and Spine Density in Layer 5 Motor Cortical Neurons of YFP-H Mice

**DOI:** 10.1371/journal.pone.0031896

**Published:** 2012-03-07

**Authors:** David P. Stuss, Jamie D. Boyd, David B. Levin, Kerry R. Delaney

**Affiliations:** 1 Department of Biology, University of Victoria, Victoria, Canada; 2 Department of Psychiatry, Faculty of Medicine, University of British Columbia, Vancouver, Canada; 3 Department of Biosystems Engineering, University of Manitoba, Winnipeg, Canada; National Institutes of Health, United States of America

## Abstract

Rett Syndrome (RTT) is a neurodevelopmental disorder predominantly caused by mutations in the X-linked gene *MECP2*. A primary feature of the syndrome is the impaired maturation and maintenance of excitatory synapses in the central nervous system (CNS). Different RTT mouse models have shown that particular *Mecp2* mutations have highly variable effects on neuronal architecture. Distinguishing MeCP2 mutant cellular phenotypes therefore demands analysis of specific mutations in well-defined neuronal subpopulations. We examined a transgenically labeled subset of cortical neurons in YFP-H mice crossed with the *Mecp2^tm1.1Jae^* mutant line. YFP^+^ Layer 5 pyramidal neurons in the motor cortex of wildtype and hemizygous mutant male mice were examined for differences in dendrite morphology and spine density. Total basal dendritic length was decreased by 18.6% due to both shorter dendrites and reduced branching proximal to the soma. Tangential dendrite lengths in the apical tuft were reduced by up to 26.6%. Spine density was reduced by 47.4% in the apical tuft and 54.5% in secondary apical dendrites, but remained unaffected in primary apical and proximal basal dendrites. We also found that MeCP2 mutation reduced the number of YFP^+^ cells in YFP-H mice by up to 72% in various cortical regions without affecting the intensity of YFP expression in individual cells. Our results support the view that the effects of MeCP2 mutation are highly context-dependent and cannot be generalized across mutation types and cell populations.

## Introduction

Rett Syndrome (RTT) is neurodevelopmental disorder primarily caused by mutations in the X-linked gene methyl-CpG-binding protein 2 (*MECP2*) [1]. RTT has broad phenotypic effects that include cognitive, motor, language, and social deficits, as well as autonomic dysregulation [2], [3]. Concordant with this wide spectrum of neurological symptoms, MeCP2 appears to regulate activity-dependent synaptic maturation and maintenance [4]–[6]. The typical RTT brain has been characterized as developmentally arrested at approximately one year of age, although this effect is highly variable [7]. Global brain volume is reduced but gross morphology is preserved, with frontal regions tending to exhibit more severe effects, which correlate with measures of clinical severity [8], [9]. Early human studies indicated that reductions in brain volume were not caused by neurodegeneration but were instead a consequence of increased neuronal density arising from reduced soma size and dendritic arborization [10]–[15].

Mapping the impact of MeCP2 mutations on the nervous system has been complicated by a high degree of phenotypic variability in both human patients and animal models. At the cellular level, the emerging perspective is that RTT neuronal phenotypes potentially depend on several interacting factors, including X-chromosome inactivation (XCI) ratio, specific mutation type, brain region, and cellular subtype. Human phenotype-genotype correlation studies have shown that both skewed XCI ratios and mutation type correlate with variations in clinical severity [16]–[19]. A similar spectrum of variability has been observed in several murine RTT models carrying different *Mecp2* mutations [20]–[24]. Neuroanatomical studies in these different mouse lines have revealed both overlapping and divergent effects on brain region volumes, neuronal density, dendritic and axonal morphology, dendritic spine density, and spine morphology [6], [25]–[34]. Both cell-autonomous and non-cell-autonomous effects have been reported and different mutations selectively affect specific morphological features while leaving others intact [29], [32]. Collectively, these studies point to a crucial role for cellular context in modulating the effects of different mutations. The use of precisely defined cellular subtypes is therefore necessary for resolving the manner in which MeCP2 mutation effects may be generalized across different cell subpopulations in the CNS.

Motor cortex is of particular interest because of the frontal volume reductions and prominent motor dysfunctions observed in RTT, which include apraxia, ataxia, repetitive stereotyped hand movements, impaired balance, and loss of ambulation [2], [35]. Early Golgi impregnation studies of neuron morphology identified dendritic branching losses in Layer 5 (L5) pyramidal cells of the motor cortex [11], [15]. L5 pyramidal neurons are not a unitary class, however, and can be grouped into subtypes based on phylogeny, gene expression profiles, morphology, electrophysiology, and axonal projection targets [36]–[41]. Since MeCP2 mutations could potentially affect any of these features, the selection of a cell population for phenotypic analysis must take these properties into account.

Transgenic labeling provides a convenient method for identifying some of these populations, as in the widely used YFP-H line (B6.Cg-Tg(Thy1-YFPH)2Jrs/J ([42] also [38], [43]–[48]). YFP-H mice express yellow fluorescent protein under the *Thy-1* promoter in a restricted set of L5 cortical neurons. In the motor-frontal cortex of YFP-H mice, YFP-expressing (YFP^+^) pyramidal neurons have electrophysiological properties and patterns of synaptic connectivity that are distinct from both neighbouring non-YFP^+^ cells and from YFP^+^ cells in other cortical regions [38], [40], [49]. We crossed YFP-H mice with the “Jaenisch” (MeCP2J) mouse line, *Mecp2^tm1.1Jae^*/Mmcd, which has an in-frame N-terminal deletion of *Mecp2* exon 3 [20]. A similar cross-breeding strategy was used for a different mutation, the protein-null *Mecp2^tm1.1Bird^* or “Bird” line (MeCP2B), in which Thy-1-GFP-labeled L5 neurons revealed significant spine losses throughout the dendritic arbor [32]. The rationale for the current study was motivated in part by the erroneous initial classification of both MeCP2B and MeCP2J mice as harboring protein-null mutations [20], [21]. In addition to our own immunohistochemical findings (unpublished data), multiple lines of evidence have emerged demonstrating that the MeCP2J line expresses a partly functional truncated MeCP2. These include the presence of stable MeCP2 mRNA transcripts, divergent gene expression profiles, and a milder phenotype in terms of brain weight, brain region volumes, and dendritic spine morphology [30], [31], [50]–[52]. In the context of these reports and previous analyses focused on L2/3 neurons in MeCP2J mice [28], [29], [31], we examined dendrite architecture and spine density in YFP^+^ L5 cells in the motor cortex of wildtype (WT) and MeCP2J mutant animals.

## Results

### YFP^+^ mutant neurons have selective reductions in dendrite length, branching, and spine density

**Figure 1:**
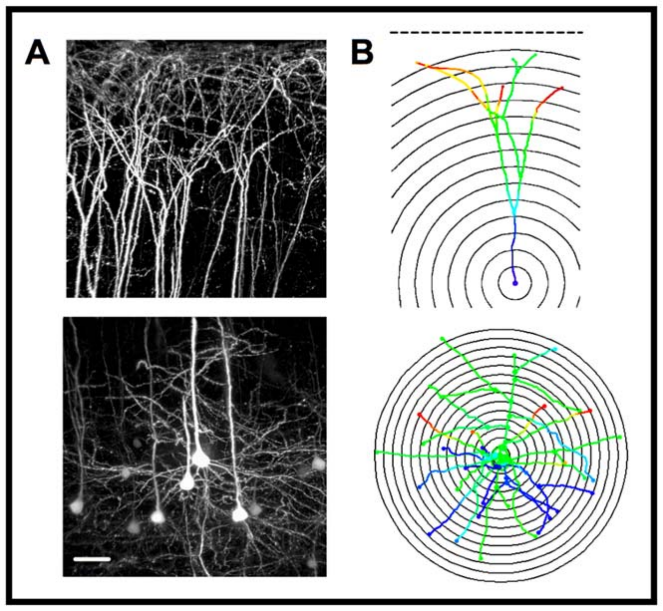
3D Sholl analysis of apical and basal arbors in YFP^+^ L5 motor cortical neurons. A) Confocal microscopic image stacks from 200 µm tissue sections showing YFP^+^ dendrites in apical (upper) and basal (lower) compartments (WT male mouse). Scale bar = 50 µm. B) Representative 3D traces from a WT neuron. 10 µm Sholl radii were used in the basal compartment and 20 µm radii in the apical compartment. The pial surface is shown as a dotted line with the apical Sholl origin defined as a point 300 µm from the pial surface. Depth in the z-axis is color-coded.

The large size of L5 pyramidal neurons and the density of YFP labeling in YFP-H mice precluded the imaging of entire cells, so 3D confocal fluorescence image stacks were obtained independently for dendrites in both the apical and basal compartments. Basal dendrite image stacks were centered on L5 YFP^+^ somata, while apical stacks were bounded by the pial surface, allowing visualization of the most distal branches of the apical tuft (Fig. 1A). Manual 3D reconstructions were made of the complete dendritic arbor in each compartment for all neurons passing exclusion criteria (Fig. 1B; *see* *Methods*).

**Figure 2:**
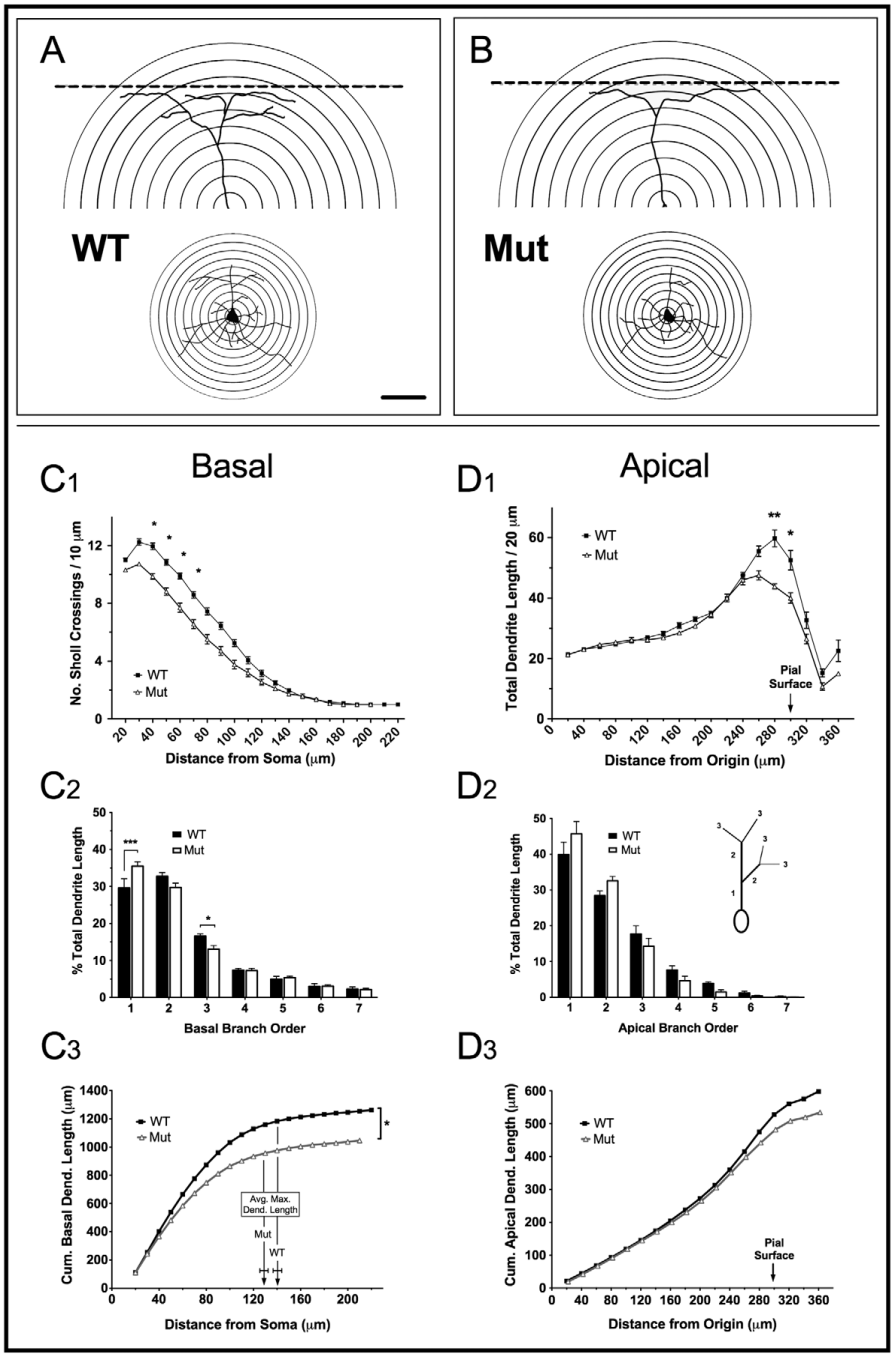
Dendritic branching and length is reduced in YFP^+^ MeCP2J mutant neurons. A, B) Reconstructions of representative WT and mutant YFP^+^ L5 pyramidal neurons based on the Sholl analysis branch patterns in C_1_ and D_1_. Scale bar (A) = 50 µm. C_1_) Basal compartment Sholl analysis showing the number of dendrite crossings as a function of distance from the soma. Mutant YFP^+^ neurons have significantly fewer dendritic branches 40–70 µm from the cell body. D_1_) Apical compartment Sholl analysis of the summed dendritic length per Sholl radius. Mutant YFP^+^ neurons have less total dendritic length per Sholl radius in L1–2. Dendrite lengths in radii greater than 300 µm correspond to long, laterally extending dendrites that are near and parallel to the pial surface. C_2_, D_2_) Percent dendritic length as a function of branch order in basal and apical compartments. The inset in (D_2_) shows branch order hierarchy. In basal dendrites only, mutant YFP^+^ neurons exhibit significantly reduced dendritic complexity with proportionally more dendritic length in lower branch orders. C_3_) A cumulative total length plot for basal dendrites shows that the main divergence in total dendritic length occurs less than ∼120 µm from the soma. The average maximum dendritic length is also reduced in mutant YFP^+^ neurons. D_3_) A cumulative total dendritic length plot of apical tuft branches shows no minimal divergence except close to the pial surface, where mutant YFP^+^ cells extend fewer lateral branches.

The largest differences we observed in YFP^+^ mutant cell morphology were in the basal arbor (Fig. 2C_1–3_). First, although very long individual dendrites were often observed in mutant neurons, the average maximum dendritic length was reduced by 9.1% (128.1 µm±3.463 in mutant vs. 140.9 µm±3.782 in WT, *t*_8_ = 2.490, *P* = 0.0375). 3D Sholl analysis revealed no difference in the number of primary dendrites in mutant neurons, but fewer bifurcations in a zone 40–70 µm from the soma (Fig. 2C_1_; one-way ANOVA, F_14,112_ = 3.908, *P*<0.001; *t*_8_>2.999, *P*<0.05). Two-way repeated measures ANOVA also revealed a significant interaction between branch order and genotype (Fig. 2C_2_; F_6,28_ = 6.849, *P* = 0.0001). Mutant neurons showed a corresponding increase in the proportion of dendrite length within the first branch order (+5.93%; *t*_6_ = 5.053, *P*<0.01), and significantly less in the third branch order (−3.5%; *t*_6_ = 2.987, *P*<0.05). Plotting the cumulative increase in total basal dendritic length vs. distance from the soma (Fig. 2C_3_) showed that the loss of higher-order branching close to the soma reduced the total dendritic length for MeCP2 mutant neurons by 18.6% (WT: 1192.0 µm±61.54; Mut 969.8 µm±65.54; *t*_8_ = 2.472, *P* = 0.0386). The largest divergence in cumulative dendritic length occured between approximately 40–130 µm from the soma. WT YFP^+^ neurons therefore maintain a larger number of dendritic branches in proximal portions of the basal arbor, as shown in reconstructions of representative “average” neurons from the Sholl analysis for each genotype (Fig. 2A–B).

In the apical compartment the Sholl analysis was modified to accommodate the fact that secondary apical dendrites can extend over long distances roughly parallel to the pial surface without crossing a Sholl radius. We therefore measured the summed dendritic lengths per 20 µm radius. The Sholl origin was arbitrarily defined as a point on the apical dendrite 300 µm from the pial surface (Fig. 1B). In motor cortex this point is situated in cortical layer 3 in both mutant and WT mice, and corresponds to a distance of ∼100–200 µm from L5A somata in the WT and slightly less in the mutant. Differences in the apical compartment were more subtle than those seen in basal dendrites (Fig. 2D_1–3_). Mutant neurons had up to 26.6% less summed dendritic length in Sholl radii 60 µm from the pial surface, corresponding to losses in the neuropil-dense cortical Layer 1 (Fig. 2D_1_; one-way ANOVA, F_16,128_ = 2.143, *P* = 0.01; Bonferroni post-hoc tests *t*_8_>3.174, *P*<0.05). This did not significantly change the total dendritic length in tuft branches (WT: 526.1 µm±28.86; Mut: 472.9 µm±22.07; *t*_8_ = 2.472, *P* = 0.1809), and we found no difference in the average maximum length of individual apical dendrites. Similarly, no difference was observed when total length analysis was restricted to apical tuft branches within less than 100 µm of the pia (not shown). Although there was an interaction between branch order and genotype (F_6,28_ = 2.547, *P* = 0.0428), with mutant neurons tending to have a larger proportion of total dendritic length in primary and secondary branches, post-hoc tests found no significant differences at any specific branch order (Fig. 2D_2_). The cumulative average dendritic lengths only diverged close to the pial surface to a final difference of 10.3% less total apical length in mutant cells, although this effect was not statistically significant (Fig. 2D_3_). Overall, these findings suggest that, in contrast to the basal compartment, the MeCP2J mutation reduces higher-order branching in YFP^+^ L5 apical tuft dendrites in a relatively non-specific manner, with some reductions of lateral dendritic branching in L1. Representative apical tuft dendrites (reconstructed from the Sholl analysis) are shown in Fig. 2A and 2B.

**Figure 3:**
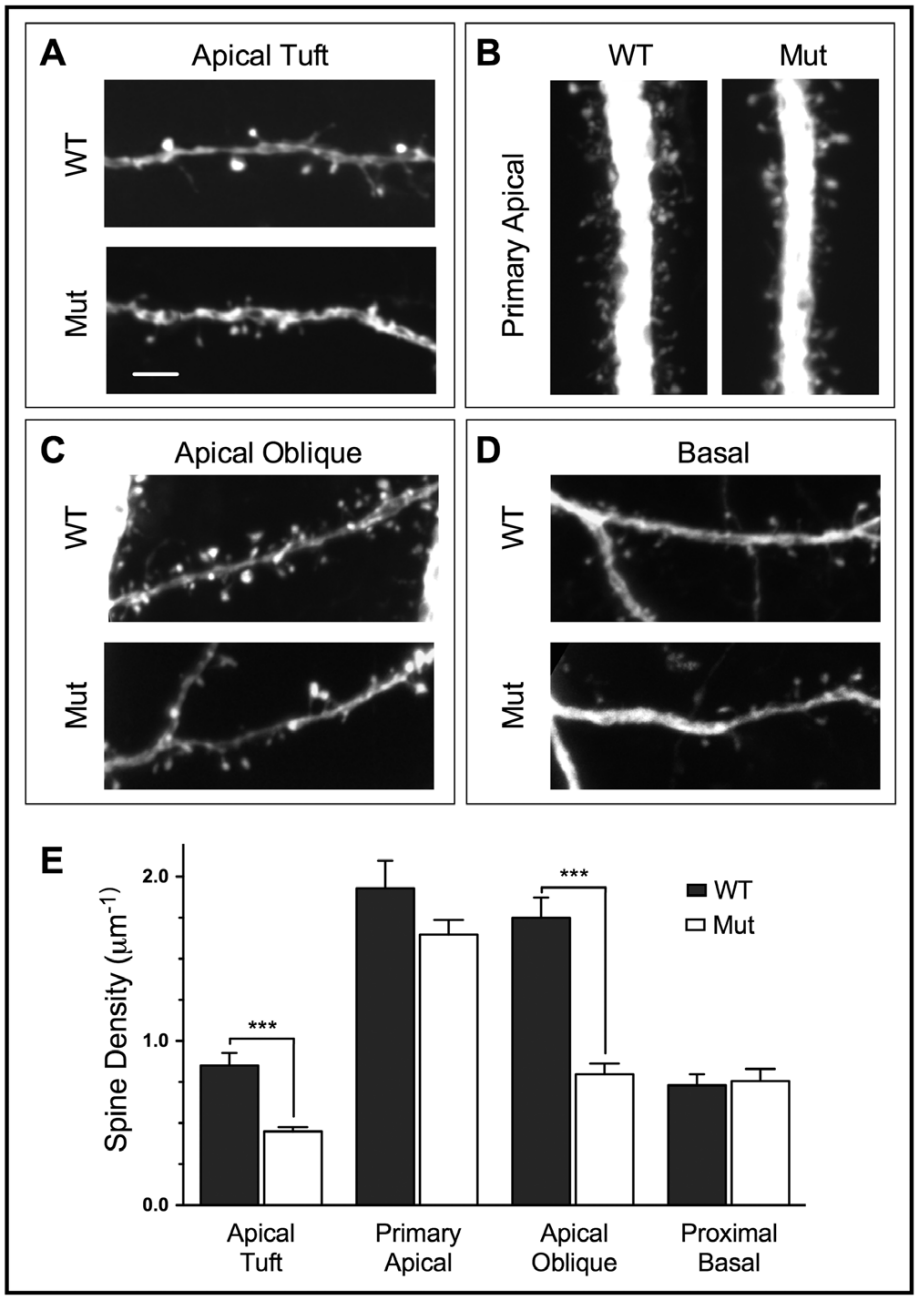
Spine density is variably affected in different dendritic compartments in YFP^+^ MeCP2J mutant L5 pyramidal neurons. A–D) Spines were quantified in 20–30 µm segments in four dendritic compartments for WT and mutant YFP^+^ neurons. Scale bar (A) = 5 µm (all images). A) Apical tuft dendrites were sampled <60 µm from the pial surface. B) Primary apical dendrites were imaged between 100–200 µm from the soma. C) Secondary apical dendrites originating in the same region as (B) were sampled beginning at the branch point from the primary apical dendrite. D) Basal dendrite spine counts were initiated following the first dendritic bifurcation. E) Spine density is reduced by 47.4% in the apical tuft and by 54.5% in secondary apical dendrites.

Any net losses in total dendritic length will reduce the total synaptic input for a given neuron, but this may be either mitigated or exacerbated by changes in dendritic spine density. To address this question we quantified the number of spines in the dendritic regions that showed the largest differences in the Sholl analyses, i.e. proximal basal dendrites and the apical tuft (Fig. 3A, 3D). Basal spine counts started after the first bifurcation (typically 20–50 µm from the soma) and apical tuft segments were not more than 60 µm from the pial surface. We also counted spines along the primary apical dendrite, 100–200 µm from the soma, and secondary oblique dendrites immediately projecting from the primary apical dendrite over this same region (Fig. 3B, 3C). Mutant neurons exhibited altered spine density, but only in some dendritic compartments (Fig. 3E). Spine density in the apical tuft was reduced by 47.4% (WT: 0.854 µm^−1^±0.0774; Mut: 0.449 µm^−1^±0.0261; *t*_23_ = 4.469, *P* = 0.0002); in secondary apical dendrites, density was reduced by 54.5% (WT: 1.751 µm^−1^±0.123; Mut: 0.797 µm^−1^±0.0657; *t*_28_ = 6.860, *P*<0.0001). In mutant primary apical and basal dendrites, however spine density remained unchanged (Primary apical, WT: 1.933 µm^−1^±0.167, Mut: 1.648 µm^−1^±0.0903; *t*_28_ = 1.623, *P* = 0.1159); (Basal, WT: 0.720 µm^−1^±0.0539, Mut: 0.756 µm^−1^±0.0733; *t*_23_ = 0.3716, *P* = 0.3716). These compartment-specific effects suggest that mutant neurons experience a significant net loss of synaptic input in the apical tuft, due to the compounded effects of both lower spine density and reduced dendritic branching.

### Thy-1-YFP expression is reduced in MeCP2J mutant mice

**Figure 4:**
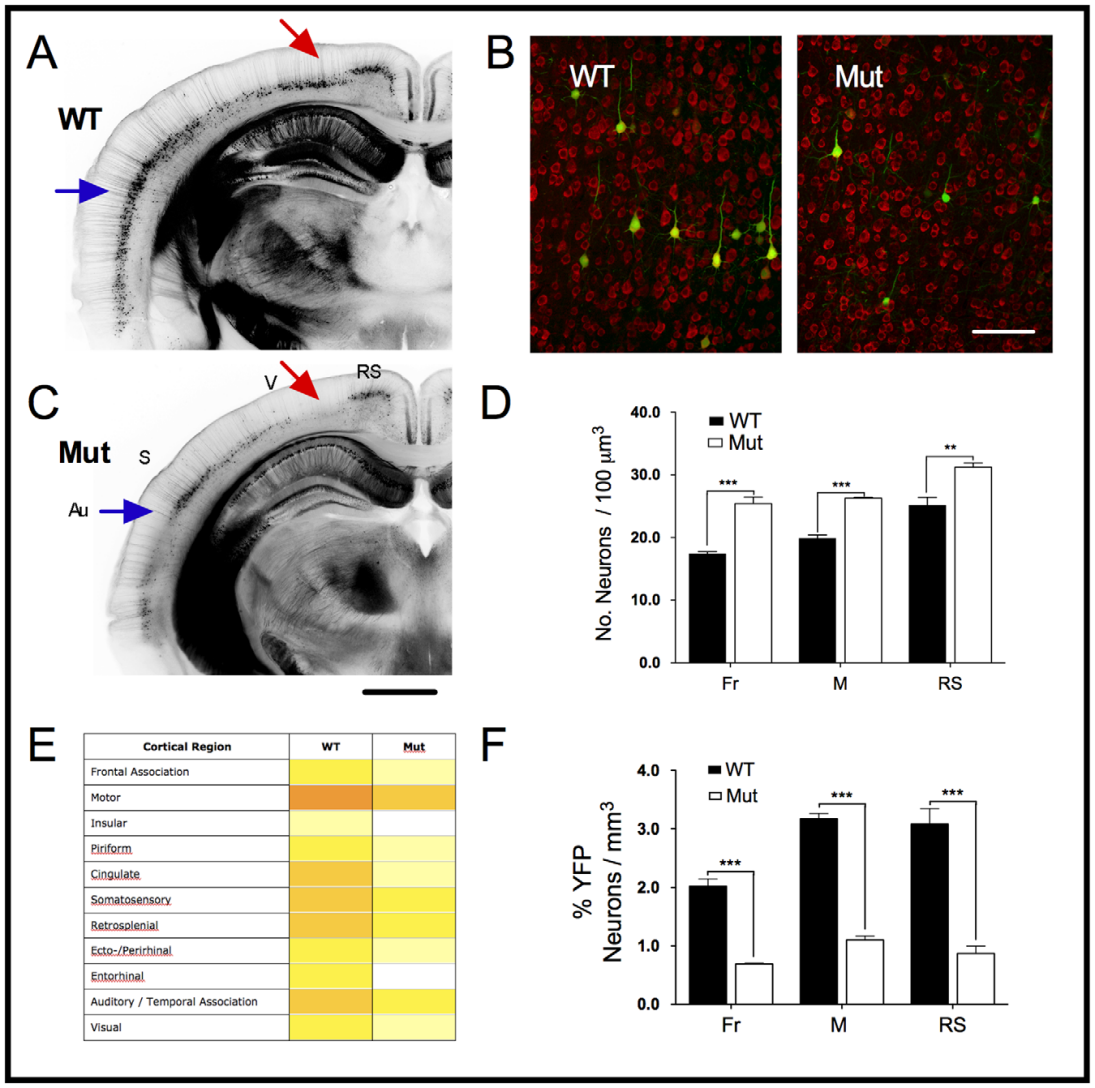
MeCP2J mutant mice have reduced numbers of YFP-expressing cortical neurons. A, C) Fewer YFP^+^ cells are observed across the entire cortex in mutant males (200 µm coronal sections, ∼ Bregma −2.2 mm in WT). Some cortical regions are more dramatically affected than others, such as visual cortex (red arrow) and auditory cortex (blue arrow). Sharp boundaries correspond to functional regions (Au, auditory; S, somatosensory; V, visual; RS, retrosplenial). Scale bar = 0.5 mm. Overall cortical YFP expression patterns are shown as a qualitative heat map in E), where white indicates few to no neurons and dark orange indicates the most dense cortical labeling. The number of YFP^+^ cells is generally higher in rostral and medial region in both genotypes. B) Fluorescent Nissl staining (red) of L5 motor cortex. A limited subset of pyramidal neurons express YFP. Scale bar = 100 µm. D) Neuronal density is increased in frontal (Fr), motor (M) and retrosplenial (RS) cortex in mutant mice. F) The percentage of YFP^+^ pyramidal neurons is reduced in three cortical regions (F, frontal; M, motor; RS, retrosplenial).

In addition to the morphological differences in MeCP2J mutant YFP^+^ neurons, we also observed an unexpected reduction in the number of YFP^+^ cells in mutant animals relative to WT littermates. To further characterize these differences we performed a qualitative survey of cortical YFP expression patterns (Fig. 4). YFP expression varied in two significant ways (summarized in Fig. 4E). First, despite some inter-litter variability, the density of labeling in both genotypes followed a similar pattern of variation across cortical regions. In some cases these differences had sharp boundaries correspondent with functional divisions of the cortex [53]. The highest densities of YFP expression tended to occur in more rostral and medial regions (e.g. motor, cingulate, and retrosplenial cortex) while the lowest densities tended to occur in caudal and lateral regions (e.g. visual, ectorhinal, entorhinal, and piriform cortex). Second, YFP density appeared to be lower in MeCP2 mutants relative to WT littermates in most cortical regions. Some regions were more dramatically affected than others, such as visual cortex, which was usually marked by an almost complete absence of YFP^+^ cells (Fig. 4A, 4C). We quantified the percentage of YFP^+^ cells in three areas (frontal association, motor, and retrosplenial cortex) as a fraction of all fluorescent Nissl-stained L5 neurons (Fig. 4B). In all three regions, mutant mice had fewer YFP^+^ neurons (one-way ANOVA, F_5,17_ = 63.36, *P*<0.0001). The fraction of YFP^+^ neurons was decreased by 65.6% in frontal cortex, 65.3% in motor cortex, and 71.8% in retrosplenial cortex (*t_5_*>6.751, *P<*0.001 in all pairs) (Fig. 4F). The reductions occurred in spite of higher neuronal density in these same regions (Fig. 4D; one-way ANOVA, F_5,12_ = 3.689, *P<*0.0001), with density increases of 46% in frontal cortex, 32% in motor cortex and 24% in retrosplenial cortex.

The reduced number of observed YFP^+^ cells could result from 1) more extensive transgene silencing in the mutant animals, with YFP^+^ neurons having similar expression levels in both genotypes or 2) overall downregulation of YFP expression levels in MeCP2 mutant animals, so that some neurons expressed YFP below detection limits.

**Figure 5:**
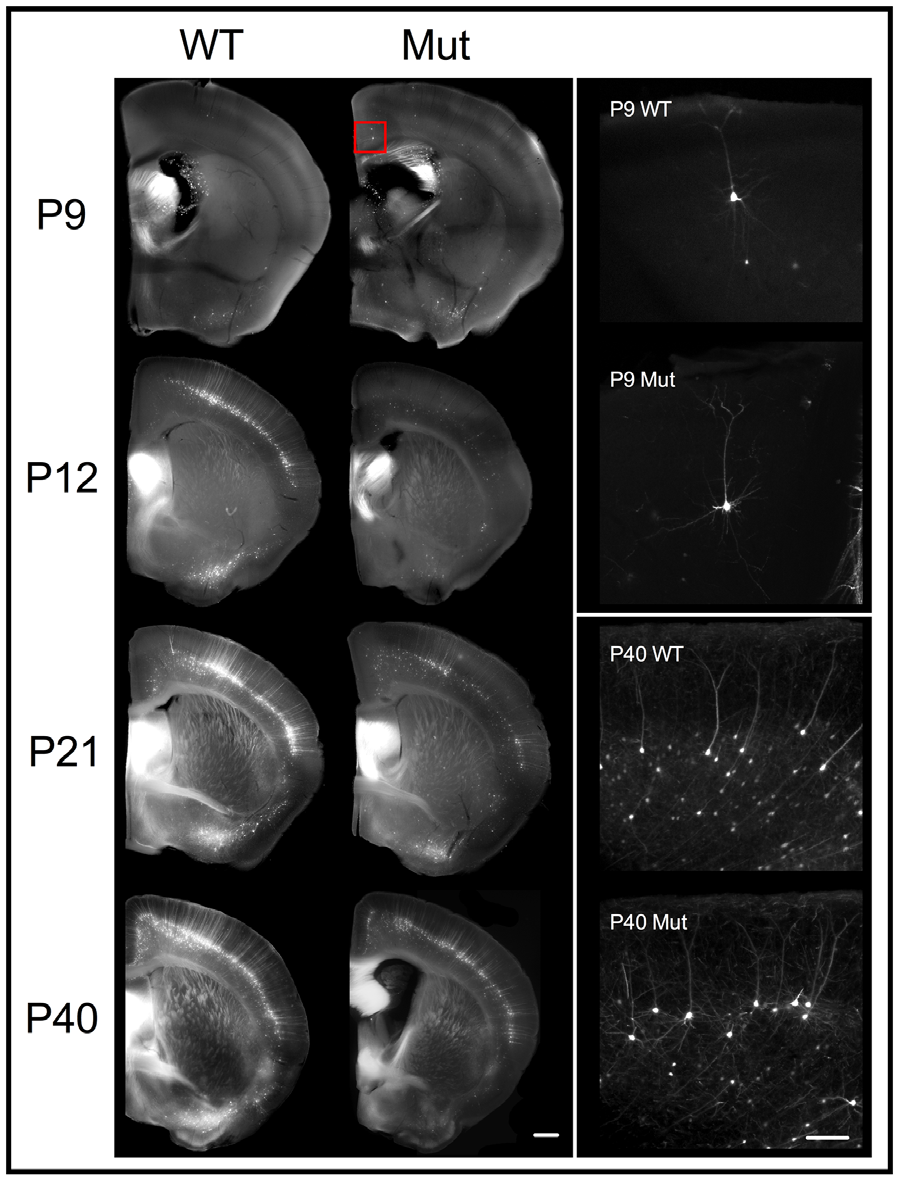
Developmental progression of cortical YFP expression. Left column: 200 µm coronal sections from YFP-H MeCP2J littermate pairs (∼ Bregma 0.14 mm in WT). The anterior commissure was used as a reference anatomical marker. Right column: maximum intensity projections of confocal fluorescence imaging z-stacks from cingulate cortex. The red box in P9 mutant (rotated 90° clockwise) corresponds to the region imaged in each brain. Single bright YFP^+^ L5 pyramidal neurons are observed in both genotypes as early as P9. By P12 YFP expression has been strongly upregulated in the WT, after which the number of YFP^+^ cells continues to increase more slowly until stabilizing to mature levels sometime between P21 and P40. The number of YFP^+^ cells in mutant males also continues to increase, but at a slower rate, and final levels remain below those seen in the WT. All confocal fluorescence images shown were obtained from the brain section shown except P9 WT, which was obtained from a tissue section 400 µm anterior due to sparse YFP labeling at this age. Left column scale bar = 0.5 mm. Right column scale bar = 100 µm.

Neuronal transgene expression under the *thy1.2* cassette begins during postnatal days P6–P10 [54], so we examined the developmental expression patterns of YFP in littermates from both genotypes at early postnatal stages to late maturity (1–18 wks) to compare these two alternatives. Representative examples are shown in Fig. 5. In both genotypes, we found large, bright individual neurons as early as P9. By P12–P14 the number of YFP^+^ cells increased sharply in WT, but remained low in MeCP2 mutants. YFP^+^ cells continued to increase in number in both genotypes until reaching stable mature levels at approximately one month of age. MeCP2 mutant mice maintained lower numbers of YFP^+^ cells at all later developmental stages. Similar confocal fluorescence imaging conditions (exposure, gain, laser intensity) were used in all cases, but increasing any or all of these parameters did not reveal any additional cells in either genotype. Furthermore, while the somata of YFP^+^ L5 pyramidal cells were 24.1% smaller in mutant male mice (WT: 181.1 µm^2^±11.1, Mut: 137.4 µm^2^±9.0; *t*_9_ = 3.097, *P* = 0.0128), there was no correlation between soma size and fluorescence intensity level in either genotype (r^2^<0.06471, d.f.>41, both genotypes; data not shown). These results suggest that the reduced number of YFP^+^ neurons in mutant mice results from transgene silencing rather than as a consequence of a higher proportion of cells expressing YFP below detection limits.

## Discussion

### Morphological Analysis

We analyzed the impact of the MeCP2J mutation on YFP^+^ L5 pyramidal cells and found two principal effects: 1) reduced branching and length in proximal basal and apical tuft dendrites, and 2) selective reductions in spine density limited to the apical tuft and secondary apical dendrites. These results contribute to a growing body of evidence showing that the effects of different MeCP2 mutations are highly sensitive to cellular context and cannot be readily generalized.

The morphological differences observed in MeCP2 mutant neurons vary both across mutation types in the same cell population and within mutation types across different cell populations. In MeCP2J mice, our secondary apical dendrite spine density values are in close agreement with those previously reported for Golgi-stained L5 motor cortical neurons [34]. By contrast, in a comparable study that used a highly similar transgenic cell population (Thy-1-GFP-labeled L5 neurons in the motor cortex), but harboring the protein-null MeCP2B mutation, spine density was significantly reduced in all dendritic compartments analyzed, including primary apical, secondary apical, and basal dendrites [32]. In L2/3 pyramidal neurons, MeCP2J mutant cells were reported to exhibit no changes in spine density, but had relatively large reductions in both apical and basal dendritic branching [28], [29], [31]. At the other phenotypic extreme, in the B6.129S-*Mecp2^tm1Hzo^*/J (MeCP2^308/Y^) mouse line, which has a C-terminal truncating deletion in MeCP2, neither dendritic branching nor spine density are affected, in both L2/3 as well as L5 cells [26].

The varied and contradictory nature of these results may be a direct consequence of the intrinsically disordered structure of MeCP2, which confers autonomous functionality to several biochemical domains that may act alone or in concert [55]. The pairing of a highly flexible tertiary structure with independently operating functional domains presents an attractive explanation for the multifunctional nature of MeCP2 as well as the diversity of mutant phenotypes. In conjunction with physiological and transcriptional profiling, direct morphological comparison between cellular subpopulations, both within and across mutant mouse lines, may present a valuable tool for dissociating how each MeCP2 domain contributes to the generation or maintenance of cellular character in the CNS.

### Interaction of Mecp2 Mutation with the Thy-1-YFP Transgene

We analyzed the effects of a specific *Mecp2* mutation on a fluorescently labeled population of cortical neurons. Similar strategies using *Thy-1-YFP/-GFP* mice have been been employed elsewhere in studies of *Mecp2* and other transgenes (e.g. [32], [56]–[60]). The principal utility of this approach arises from the use of cell type-specific promoters to restrict analysis to defined cellular subtypes. Given the increasingly evident taxonomic diversity of neuronal subpopulations based on clustered patterns of hodology, morphology, gene expression, and electrophysiological properties [61]–[63], this type of approach is most salient when evaluating gene mutations with highly context-dependent effects.

The diverse and often contradictory effects of MeCP2 mutation are not surprising given its complex regulatory network and multifunctional capacity. Expression levels of the two MeCP2 isoforms are non-homogeneous and vary with brain region and cortical layer [22], [64]–[67]. MeCP2 is also subject to multiple transcriptional, post-transcriptional, and post-translational regulatory influences [68]–[72]. Furthermore, the full functional repertoire of MeCP2 remains incompletely understood, but at present includes chromatin remodelling, gene silencing, transcriptional modulation, RNA splicing, and regulation of microRNAs [73]–[78]. Transcriptional regulation alone differentially affects thousands of genes and varies by neuroanatomical region [79]–[82]. Selectively labeled subpopulations like those in the YFP-H line present an attractive means to address questions about the context-dependent effects of different MeCP2 mutations.

However, this advantage is complicated by the potential for interactions between the mutant protein and the reporter transgene. We found that in MeCP2J mutant male mice, the number of YFP-expressing cells was decreased by 65–70% in several brain regions, while the intensity of YFP fluorescence in individual cells was preserved. More extensive *Thy-1-YFP* silencing in mutant mice would appear at odds with the role of MeCP2 as a transcriptional repressor [73], [83], but MeCP2 does influence the expression of other transcriptional regulators [84]. Furthermore, the *Thy-1* promoter expression cassette is highly sensitive to chromosomal context, and identical constructs can produce highly variable expression patterns across transgenic lines [42], [54]. To date, the *Thy-1* gene has not emerged as significantly altered in any gene profiling studies, but effects on *Thy-1* promoter-driven transgenes are not entirely surprising given the involvement of MeCP2 in chromatin remodelling. The sharply delineated, region-dependent decreases in YFP^+^ cell numbers that we observed in some cortical areas suggests that these changes are not a random consequence of altered chromatin architecture, but may instead reflect distinct changes in regulatory pathways for different cell populations.

Our study does not rule out the possibility that the reduced number of YFP^+^ cells in MeCP2J mutants correlates with a selective alteration in the proportions of certain subclasses of Layer 5 pyramidal neurons. Although variegation between founders in transgenic mouse lines has been considered a stochastic effect arising from random chromosome insertion site and transgene copy number [85], [86], several reports suggest that the pattern of YFP^+^ pyramidal cells in the YFP-H line is not random, but constitutes a limited set of distinct functional subclasses defined in part by their principal axonal targets [38], [40], [49]. Intriguingly, axonal targeting is disordered in MeCP2 mutant mice [31], [33] and the developmental timing of axonal pruning partially overlaps with that of MeCP2 expression in cortex [22], [66], [87]–[91]. Establishing how the MeCP2J mutation affects factors driving *Thy-1-YFP* expression could reveal new roles for distinct functional domains of MeCP2 in establishing or maintaining subtle differences between closely related neuronal subpopulations.

## Methods

### Ethics Statement

All procedures performed on animals were in compliance with the Canadian Council on Animal Care guidelines and were approved by the University of Victoria Animal Care Committee (Approval number 2008-021).

### Animal Breeding

*Mecp2* mutant mice (*Mecp2^tm1.1Jae^/*Mmcd) (MMRRC, UC Davis) [20] were maintained on a 129S2/SvPasCrl background (Charles River) and YFP-H transgenic mice (B6.Cg-Tg(Thy1-YFPH)2Jrs/J, Jackson Laboratory) [42] were independently maintained on a C57BL/6J background. *Mecp2* heterozygous female mice were crossed with YFP-H male mice homozygous for the YFP transgene to generate experimental F1 animals. *Mecp2* and *YFP* were genotyped by PCR of genomic DNA extracted from earclips. *Mecp2* genotype was determined using primer sequences [5′- CAC CAC AGA AGT ACT ATG ATC] and [ATG CTG ACA AGC TTT CTT CTA-3′] which detect wildtype and mutant *Mecp2* alleles as 3 kb and 250 bp bands respectively. Presence of the YFP transgene was determined using primers [5′- TCT GAG TGG CAA AGG ACC TTA GG] and [CGC TGA ACT TGT GGC CGT TTA CG-3′], detecting a 300 bp band.

### Histological Preparation

Animals were anaesthetized using urethane (2 g/kg i.p.) (Sigma) and transcardially perfused with 10 ml 0.1 M phosphate-buffered saline (PBS, pH 7.4) and 40 ml room-temperature 4% formaldehyde in 0.1 M phosphate buffer (PFA, pH 7.4). Brain tissue was dissected out and fixed in 4% PFA at 4°C overnight, washed three times in PBS, and sunk in 30% sucrose-PBS. Agar-embedded 200 µm coronal sections were cut on a Pelco Vibratome 1000 and coverslipped with Shandon Immunomount (Thermo Scientific).

### Imaging

1024×1024 pixel 3D confocal fluorescence image stacks were obtained using a Nikon Eclipse TE2000-U confocal microscope and Nikon EZ-C1 3.60 software or an Olympus Fluoview FV1000 with Fluoview software (v. 1.7c) and 488, 515 or 543 nm lasers. Multi-channel imaging was done sequentially. All images were averaged twice with scan speeds of 1.68–3.94 µs/pixel unless otherwise indicated. Image stacks were converted to 16-bit .tif files using ImageJ software for further analysis.

Images for neuron density and percentage of YFP^+^ cells were obtained using a Nikon Plan APO NA 0.95 20× air objective, 10×1 µm z-step and 30 µm pinhole. For reconstructions of apical and basal compartments of YFP-H neurons, images were obtained using a Nikon 40×/1.3 NA S Fluor oil immersion objective, 400 nm z-step, and 30 µm pinhole. Laser power and gain were manually adjusted during imaging to maintain maximal subsaturation pixel intensities at the apical dendrite. For dendritic spine counts of the apical and basal dendrites of YFP^+^ neurons, images were obtained using an Olympus UPlanFL N 100×/1.3 NA oil immersion objective, 5× zoom, 200 nm z-step, and 190 µm pinhole.

For qualitative analysis of YFP expression patterns, 1600×1200 pixel widefield images of cerebral hemispheres were obtained using a 4×/0.13 NA objective with a 0.5× projection lens on an Olympus IX70 inverted epifluorescence microscope. Images were captured with a Retiga 2000R digital CCD camera (QImaging). For the developmental staging of YFP expression, 2560×1920 pixel images were obtained using a Nikon 4×/0.13 NA Plan Fluor objective with a Nikon DS-U1 camera and ACT-2U software. Images were adjusted for brightness and contrast and tiled using Adobe Photoshop CS4.

### Histology & Morphological Analysis

L5 neuron density was measured by staining 200 µm coronal tissue sections with the neuron-specific fluorescent Nissl stain Neurotrace 530/615 (Invitrogen, 1∶300 dilution).

The upper boundary of L5 was determined with reference to the YFP^+^ cell somata. Only superficial (<40 µm depth) tissue was imaged due to limited dye penetration. Counts from five tissue sections per animal were averaged for each brain region analyzed. Confocal image stacks were flattened into maximum intensity projections (MIPs) prior to cell counts using the ImageJ Cell Counter plugin (Kurt De Vos, University of Sheffield). Cells occluded by the image boundary or lacking a nucleus clearly delineated by the Nissl stain were not counted, to eliminate partially imaged cells from above and below the planes included in the MIP. For YFP cell counts, YFP^+^ cells whose Nissl stains did not fit these criteria were similarly excluded.

Qualitative analysis of YFP expression patterns in the cortex used five WT/mutant littermate pairs (11–13 wks). Entire brains were cut in 200 µm coronal sections (excluding the olfactory bulbs and cerebellum) and serially mounted. Due to the marked volume reduction in the mutant mouse brains relative to WT, rostrocaudal position was established with reference to distinctive anatomical features visible in the coronal plane instead of Bregma, with reference to coronal plates in the Paxinos Mouse Brain Atlas [53]. These included: fusion of the medial orbital cortex to the anterior olfactory bulb; disappearance of the rhinal fissure; fusion of the genu of the corpus callosum; emergence of the anterior commissure and third ventricle; appearance of the dentate gyrus; and appearance and separation of the medial mammillary nucleus. After establishing the range of tissue sections over which a cortical region occurred each region was assigned a score from 0 to 4 as a qualitative estimate of the number of large YFP^+^ pyramidal cells. A score of 0 reflected very few or no cells and 4 indicated very dense labeling. A median value of the score for each cortical region was taken from all mice within each genotype and plotted using a heat map, where white represents few to no cells and dark orange represents the highest observed density of YFP-expressing neurons.

Soma traces and dendritic analyses were performed on five F1 WT/mutant littermate pairs (8–13 wks). Neuron reconstructions and soma traces were performed by a single observer blind to genotype. All 3D confocal image stacks of motor cortex were obtained from coronal tissue sections rostrally bounded by the first appearance of the corpus callosum and caudally by appearance of the anterior commissure, corresponding to a rostrocaudal depth of ∼2 mm.

Soma traces were performed using ImageJ. YFP^+^ somata were outlined in the focal plane where each cell had its maximum in-focus area. Partially occluded cell bodies at the image boundaries were not used. Prior to outlining each cell, the image was zoomed 12×. Post-hoc analysis of the mean signal∶background ratio defining the cell boundary was determined by examining 20 neurons randomly selected from previous traces in two YHM littermates. The average YFP fluorescence intensity at the cell boundary was 7.8× background intensity and not less than 3×.

Apical and basal dendrite tracings were performed on confocal image stacks using Igor Pro 6.05 software (Wavemetrics) with the Migor 4 custom analysis plugin developed by Jamie Boyd (UBC). 3D traces of apical or basal compartments were generated in Migor using a Cintiq 21 UX interactive pen display (Wacom). Apical and basal compartments were separately imaged to allow adequate resolution of fine dendritic branches in YFP^+^ neurons. For basal compartments, image stacks were oriented such that somata of YFP^+^ Layer 5 pyramidal neurons were centered along the y- axis, to permit capture of the maximum length of dendritic branching possible, as well as a minimum of 100 µm of the apical dendrite. Somata were selected from a sub-volume of the z-stack defined by a zone of exclusion comprising ∼100 µm from the outer edges of the x and y axes and the central 100 µm within the z-axis. At 40× magnification the field of view in the x-y axes was 318 µm^2^. Due to variable shrinkage in the z-axis during the mounting process the excluded z-axis guard zones were of variable length but typically ∼25 µm. Traces of the first 100 µm of the primary apical dendrite (and any secondary branches) were included as part of the basal compartment. For apical compartment traces, the pial surface of the brain was aligned parallel and contiguous with the upper boundary of the x-axis in the image plane, permitting capture of apical dendrite into deep Layer 3. A standard reference point for comparing dendritic structures was established by initiating all traces 300 µm from the pial surface. Traces were not included if obvious truncations of lateral branches occurred.

Statistical analysis of the 3D Sholl data from individual neuron reconstructions used a repeated measures ANOVA with genotype and distance from origin as predictor variables and number of dendrite crossings per Sholl radius (basal) or summed dendrite length per Sholl radius (apical) as the response variables. Differences at specific radii were analysed using Bonferroni post-hoc *t*-tests. In the basal compartment, 3D Sholl cross analysis was performed by counting the number of dendrites intersecting concentric spherical radii at 10 µm intervals. In the apical compartment, Sholl lengths were measured by summing all of the segments of dendrite length occurring within each 20 µm Sholl radius. Maximum dendritic length was defined as the largest Sholl radius crossed by at least one dendritic segment. Total cumulative dendrite length measurements were the summed length of all dendrites in each of the basal and apical compartments. Percent dendritic length was calculated as a function of branch order in each compartment. Branch order was defined by the number of branch nodes occurring following a given primary node, such that dendrites with fewer branches would have proportionally more branch length in lower branch orders.

Dendritic spine counts were performed using confocal fluorescence image stacks up to 10 µm deep in the z-axis. Image stacks were smoothed using a 2.0 pixel median filter prior to analysis. Spine counts were obtained from 20–30 µm dendritic segments of randomly selected neurons using the ImageJ Cell Counter plugin. Spines projecting directly above and below the dendrite in the z-axis were not counted. In regions of high spine density (e.g. WT primary apical) potentially overlapping spines were identified by vertically scrolling through confocal z-stacks. Due to the difficulty of unequivocally distinguishing filopodia from long thin spines, spine counts included all types of dendritic protrusions. Tissue sections were obtained from three WT/mutant littermate pairs (11–13 wks). 15–20 cells were analyzed per animal, with total spine counts of 1641 (WT) and 1440 (mutant). Spine density was calculated by dividing the total spine count by the length of dendrite analyzed. Representative images in Fig. 3 were rotated, cropped and adjusted for brightness and contrast.

### Statistical Analysis

Statistics were performed using the Migor 4 plugin (Jamie Boyd, UBC) for Igor Pro (Wavemetrics), Prism 5 v.5.0b (Graphpad Software) and R v.2.9.2 (WU). Unless otherwise stated, all parameters were analyzed using the two-tailed unpaired student's t-test and results considered significant at P<0.05, with measurements reported as mean ± SEM. Bonferroni's Multiple Comparison Test was used for pairwise comparisons for all ANOVAs. Dataset distributions were tested for normality using D'Agostino & Pearson omnibus normality test. Figures were prepared using Prism 5, ImageJ, and Photoshop CS3 v.10.0 (Adobe; San Jose, CA).
